# The cytoprotective co-chaperone, AtBAG4, supports increased nodulation and seed protein content in chickpea without yield penalty

**DOI:** 10.1038/s41598-023-45771-3

**Published:** 2023-10-29

**Authors:** Nipuni Thanthrige, Grace Weston-Olliver, Sudipta Das Bhowmik, Johannes Friedl, David Rowlings, Mehdi Kabbage, Brett J. Ferguson, Sagadevan Mundree, Brett Williams

**Affiliations:** 1 Centre for Agriculture and the Bioeconomy, Queensland University of Technology, Brisbane, QLD Australia; 2 School of Biology and Environmental Science, Queensland University of Technology, Brisbane, QLD Australia; 3 School of Agriculture and Food Sciences, University of Queensland, Brisbane, QLD Australia; 4 Department of Plant Pathology, University of Wisconsin-Madison, Madison, WI USA; 5 Department of Forest and Soil Sciences, Institute of Soil Research, University of Natural Resources and Life Sciences Vienna, Vienna, Austria

**Keywords:** Plant sciences, Plant biotechnology, Plant molecular biology

## Abstract

Drought and extreme temperatures significantly limit chickpea productivity worldwide. The regulation of plant programmed cell death pathways is emerging as a key component of plant stress responses to maintain homeostasis at the cellular-level and a potential target for crop improvement against environmental stresses. *Arabidopsis thaliana* Bcl-2 associated athanogene 4 (AtBAG4) is a cytoprotective co-chaperone that is linked to plant responses to environmental stress. Here, we investigate whether exogenous expression of *AtBAG4* impacts nodulation and nitrogen fixation. Transgenic chickpea lines expressing *AtBAG4* are more drought tolerant and produce higher yields under drought stress. Furthermore, *AtBAG4* expression supports higher nodulation, photosynthetic levels, nitrogen fixation and seed nitrogen content under well-watered conditions when the plants were inoculated with *Mesorhizobium ciceri*. Together, our findings illustrate the potential use of cytoprotective chaperones to improve crop performance at least in the greenhouse in future uncertain climates with little to no risk to yield under well-watered and water-deficient conditions.

## Introduction

Chickpea (*Cicer arietinum* L.) is the world’s third most important grain legume with a global value of approximately 4.7 billion USD annually and an excellent source of plant-based protein, minerals, vitamins, and dietary fibre^1^. Drought and extreme temperatures are the primary limiting factors in chickpea production causing up to 50% yield losses^2,3^. Chickpeas like other legumes serve as good source of high-quality food and feed. Also, they fix their own nitrogen, improving soil fertility, and reducing nitrogen fertiliser application which helps to reduce greenhouse gas emissions^4^. The capacity of legumes to fix nitrogen is due to their symbiotic relationship with rhizobia bacteria. In specialised root organs, termed nodules, the rhizobacteria fix atmospheric nitrogen (N_2_) into forms available to the host plant. In return, the plant host provides energy resources for the rhizobia, primarily through photosynthesis^5^. Due to the health and environmental benefits of consuming and growing legumes, researchers have investigated strategies to improve legume production.

Plant biotechnology, including the generation of transgenic plants, presents significant opportunities for improving crop stress tolerance. Despite considerable research efforts, many strategies fail in the field due to the multigenic nature of plant stress responses and the compounding challenge of simultaneous stresses such as heat, salinity, and drought^6,7^. One attractive approach for engineering broad-range stress tolerance in crops in the field is the regulation of programmed cell death and expression of cell death suppressors^8–13^.

Programmed cell death (PCD) is a genetically-regulated mechanism that controls cell demise in all eukaryotes and occurs in many forms^14^. The genetic regulators of apoptosis, the most well-studied form of PCD in animals, including pro and anti-apoptotic proteins, are conserved in yeasts, nematodes, and animals. Although, the conservation of apoptosis in plants is less certain, plant PCD is observed and researchers have studied the effects of animal cell death regulators in transgenic plants^9–12,15^. Transgenic plants expressing animal cell death suppressors (*Bcl-2, Bcl-xL, CED-9, Op-IAP*) are tolerant to multiple abiotic stresses and resistant against numerous biotic challenges^9–13,15^.

Accordingly, to improve the salinity tolerance of soybean (*Glycine max* L.), Robert et al. expressed the nematode cell death inhibitor *CED-9*^16^. The transgenic plants displayed superior salinity tolerance, but reduced nodulation upon rhizobia inoculation^16^. Robert et al. postulated that *CED-9* inhibits nodule organogenesis by interacting with vesicular trafficking and plant autophagy proteins^16^. Due to the poor nodulation phenotype, it was suggested that the expression of animal PCD suppressors such as *CED-9* may be an unsuitable strategy to increase the stress tolerance of nitrogen fixing legume crops^16^.

The B cell lymphoma 2 (Bcl-2)-associated athanogene (BAG) family of co-chaperones is a multifunctional group of evolutionarily conserved proteins that participate in diverse cellular functions including stress responses, proliferation, migration, and cell death^14,17^. In *Arabidopsis thaliana* seven genes encode BAG proteins, among them, *Arabidopsis thaliana BAG4* (*AtBAG4*) demonstrates the most potential for crop improvement^8,14,17,18^. Transgenic expression of *AtBAG4* in tobacco, rice, banana, and cotton improves abiotic stress tolerance including drought and suppresses plant PCD^14,18–20^. Recent research by Locascio et al. also demonstrated a role for AtBAG4 in ion flux control and stomatal movement via regulation of KAT1 potassium channels^21^. Research in mammalian systems demonstrates a role for BAG proteins within autophagy^22–24^. Before assessing the suitability of transgenically expressing *AtBAG4* as a strategy for improving chickpea cultivation, it is important to investigate the implications of *AtBAG4* on chickpea nodulation in well-watered conditions.

Here, we show that transgenic chickpeas expressing *AtBAG4* are more drought tolerant and produce higher yields under stress than non-transgenic controls. *Mesorhizobium ciceri* inoculated chickpea expressing *AtBAG4* form equivalent or more nodules compared to non-transgenic control plants. The additional nodules translated to increased shoot nitrogen content for several lines. Moreover, we observed no detrimental effect of *AtBAG4* expression on seed yield under irrigated conditions. Taken together, our findings suggest that *AtBAG4* is a suitable candidate gene to improve drought stress tolerance in chickpea without yield penalty under irrigated conditions. Furthermore, unlike *CED-9*, the expression of *AtBAG4* does not detrimentally affect nodule formation or nitrogen fixation under irrigated conditions.

## Results

### Transgenic plants overexpressing *AtBAG4* are water-deficit stress tolerant

**Figure 1:**
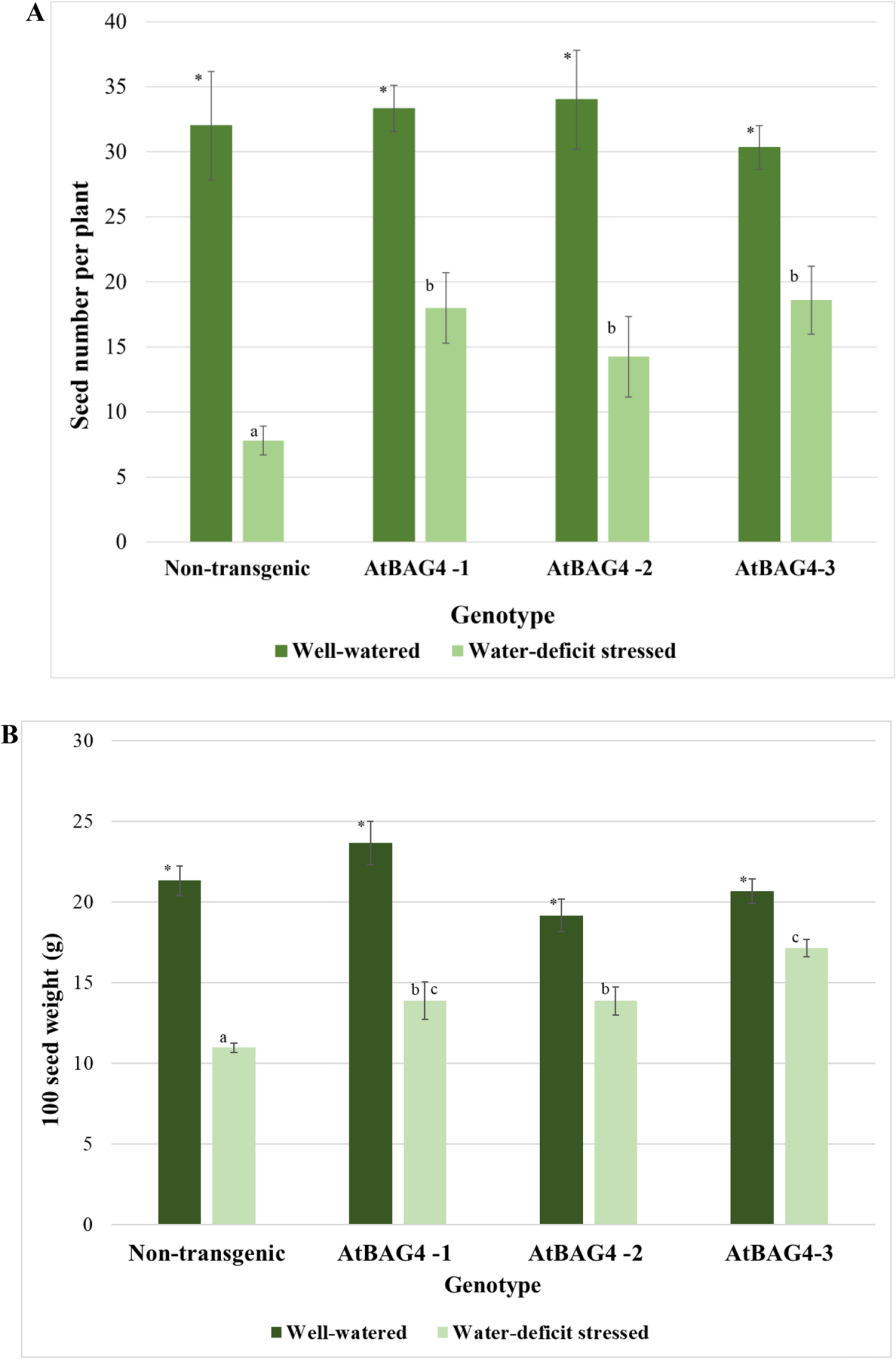
Transgenic expression of *AtBAG4* improved water-deficit stress tolerance in the greenhouse. (**A**) Number of seeds per plant under well-watered and water-deficient conditions. (**B**) Hundred seed weight of each genotype under well-watered and water-deficit stress conditions. Non-transgenic (HatTrick) and three transgenic chickpea lines (AtBAG4-1, AtBAG4-2, AtBAG4-3) were grown in the glasshouse to the flowering stage and then subjected to water-deficit stress. For the well-watered controls, the plants were watered every second day. Error bars indicate the standard error. Different letters and * represent statistically significant differences determined with ANOVA followed by Tukey’s test between transgenic and non-transgenic plants (n = 3, p ≤ 0.05).

In the field, plants are subjected to multiple stresses, simultaneously. Previous studies show that transgenic expression of cell death suppressors confers broad-spectrum stress tolerance in plants^9–13,15^. Expression of *AtBAG4* provides UV irradiation, oxidants, drought, salinity and cold tolerance in tobacco, rice, and banana^18–20^. To determine whether *AtBAG4* expression supported increased seed number per plant in chickpea, transgenic chickpea expressing *AtBAG4* and non-transgenic control plants were grown until the flowering stage and subjected to water-deficit stress. As shown in Fig. 1, the total number of seeds per plant and the 100 seed weight of the transgenic lines were significantly higher than the non-transgenic controls under water-deficit stress (Fig. 1). When well-watered, the transgenic lines performed as well as the non-transgenic controls and the number of seeds per plant and 100 seed weight was not significantly different between the transgenic and non-transgenic lines (Fig. 1). Thus, no apparent metabolic cost was observed when expressing *AtBAG4* in chickpea.

### *AtBAG4*-expressing transgenic plants nodulate at higher or similar levels compared to the non-transgenic controls upon *M*. *ciceri* inoculation

Previous studies showed that expression of the cell death suppressor *CED-9* improved *Glycine max* L. salinity tolerance but inhibited *Bradyrhizobium japonicu*m nodulation^16^. To determine the effects of transgenic *AtBAG4* on nodule formation, we inoculated four transgenic chickpea lines (AtBAG4-1, AtBAG4-2, AtBAG4-3, AtBAG4-4) expressing *AtBAG4* with *M. ciceri* and compared the number of nodules per plant, on a dry weight basis, against non-transgenic controls. Transgenic AtBAG4-3 (306 nodules per plant) and AtBAG4-4 (217 nodules per plant) lines showed significantly more nodules than the non-transgenic controls (109 nodules per plant). Transgenic AtBAG4-1 and AtBAG4-2 produced an equivalent number of nodules to the non-transgenic controls (Supplementary Fig. 1). As expected, the non-inoculated controls did not nodulate.

**Figure 2:**
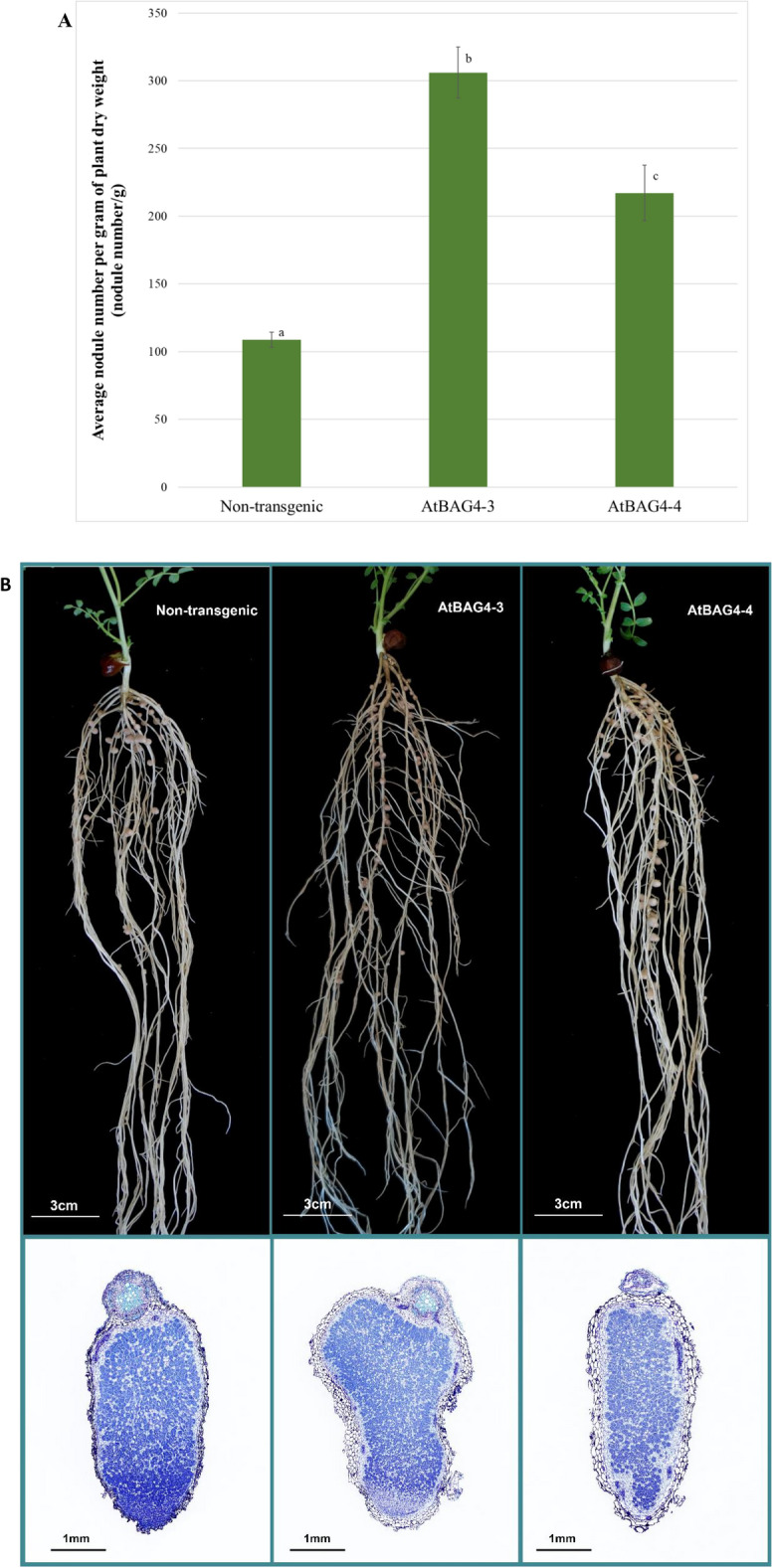
Transgenic lines expressing *AtBAG4* showed higher nodulation compared to the non-transgenic control under well-watered conditions. (**A**) Average nodule number per plant in transgenic and non-transgenic chickpeas. Error bars indicate the standard error. Different letters represent statistically significant differences determined with ANOVA followed by Tukey’s test between transgenic and non-transgenic plants (n = 5, p ≤ 0.05). (**B**) Nodule morphology of transgenic and non-transgenic chickpeas.

We then determined whether nodulation correlated with *AtBAG4* expression by quantitative real-time reverse transcription PCR (qRT-PCR) analysis. The expression of *AtBAG4* varied in the different chickpea lines (Supplementary Fig. 2). *AtBAG4* expression in the *AtBAG4*-3 transgenic line correlated with the water-deficit stress tolerance and higher nodule number per plant. However, this was not true for the other transgenic lines (Figs. 1, 2 and Supplementary Figs. 1, 2). The difference between phenotype and *AtBAG4* expression levels may be due to the T-DNA insertion site or that transcript levels do not always translate at the protein level because of post-transcriptional regulation.

Nodule morphology was similar in the transgenic lines and the controls (Fig. 2B). For all plants, nodule formation occurred primarily on the upper part of the root system, consistent with a properly functioning autoregulation of nodulation (AON) system^25^, and the nodules were pink in colour, indicating the presence of leghaemoglobin. To determine whether *AtBAG4* expression affected rhizobial colonisation, we cross-sectioned the nodules for observation under a light microscope (Fig. 2B). Infected cells were densely packed within the infection zone and we observed no clear differences between non-transgenic and transgenic chickpea nodules (Fig. 2B). We conclude that the transgenic chickpeas expressing *AtBAG4* develop morphologically equivalent nodules to the non-transgenic controls.

### *M*.* ciceri*-inoculated transgenic chickpeas expressing *AtBAG4* fix more nitrogen and contain high seed protein contents than the non-transgenic controls

Since AtBAG4-3 and AtBAG4-4 displayed improved nodulation, these two lines were used for further investigation to determine whether the increased nodulation equated to increased fixation and nitrogen content.

**Figure 3:**
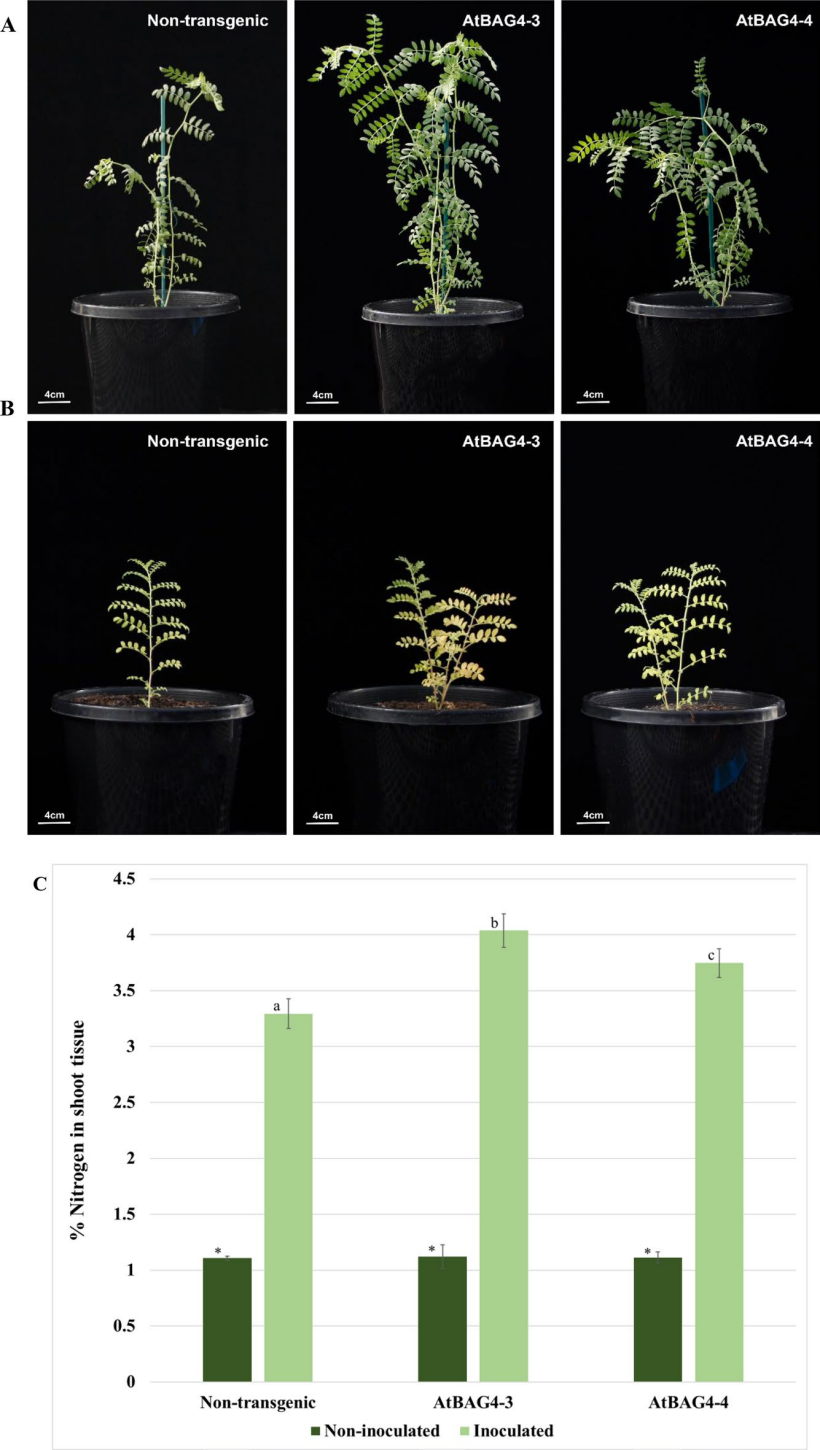

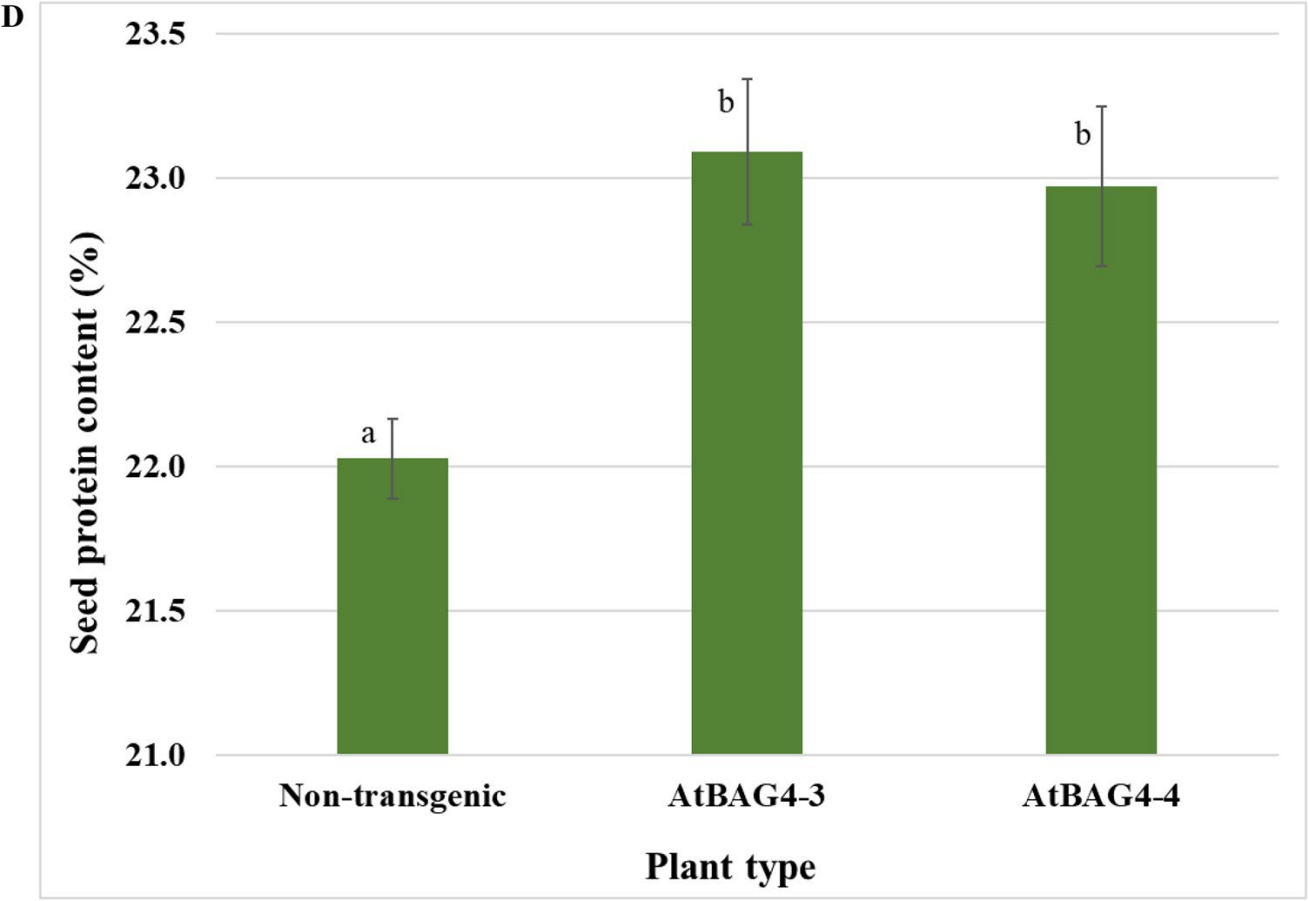
Transgenic *AtBAG4* expressing chickpea display enhanced shoot growth and accumulate more nitrogen. (**A**) and (**B**) Plant morphology of inoculated and non-inoculated chickpea plants respectively. (**C**) Nitrogen content in shoot tissue. (**D**) Seed protein content. Error bars indicate the standard error. Different letters and * represent statistically significant differences determined with ANOVA followed by Tukey’s test between transgenic and non-transgenic plants (n = 5, p ≤ 0.05).

Transgenic and non-transgenic plants were inoculated with *M. ciceri* and the shoot of each plant was harvested, dried, weighed and ground into a powder. A subsample of the powder was then used to analyse the delta ^15^N signature of the shoot using IRMS. Nitrogenase enzyme discriminates between N isotopes favouring ^14^N over ^15^N. Therefore, N fixing systems are enriched for ^14^N. All inoculated plants had negative delta ^15^N in the shoot biomass to demonstrate active nitrogen fixation and likely reflect the effects of this positive discrimination of nitrogenase for ^14^N and/or and discrimination effects during the translocation of fixed nitrogen to the shoot^26,27^ (Supplementary Fig. 3). Next, we evaluated the nitrogen yield in these plants. AtBAG4-3 and AtBAG4-4 contained statistically higher nitrogen yield (4.03% and 3.7% respectively) compared to the non-transgenic controls (3.3%) upon *M. ciceri* inoculation (Fig. 3C). We observed a significant difference between the morphology of the inoculated and non-inoculated chickpea plants. Non-inoculated plants displayed symptoms of nitrogen deficiency, including leaf yellowing and stunted growth whereas inoculated plants were green, attaining typical plant height (Fig. 3A,B). The transgenic plants expressing *AtBAG4* performed better than the non-transgenic controls under nitrogen stress, consistent with a possible role of BAG4 in autophagic protein turnover and nitrogen efficiency. Furthermore, the seed N content of the transgenic chickpea lines is significantly higher than non-transgenic controls (Fig. 3D).

### Transgenic chickpea showed higher photosynthetic rates upon inoculation with *M*. *ciceri*

**Figure 4:**
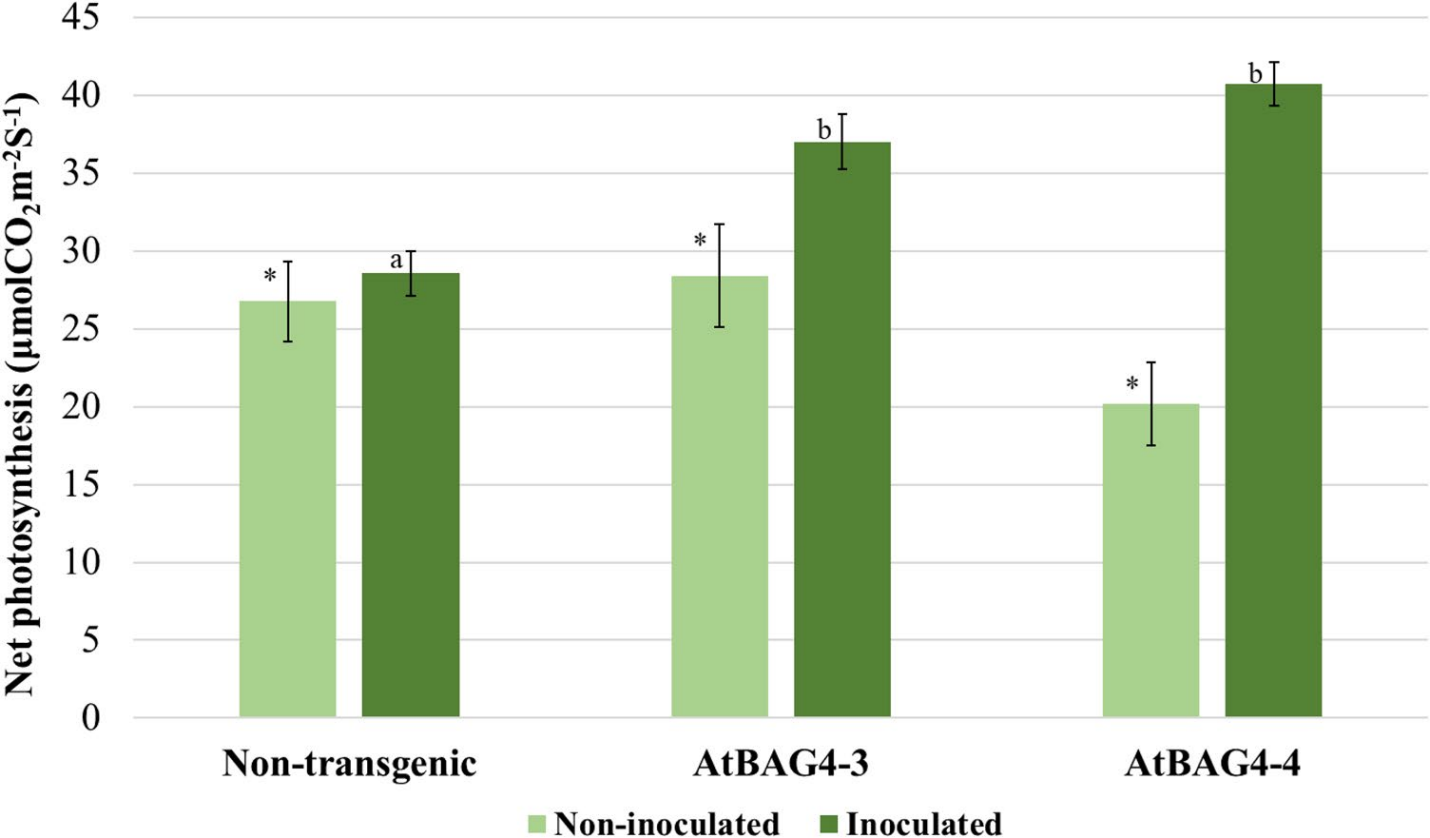
Rhizobia inoculated transgenic chickpea expressing *AtBAG4* support higher net photosynthesis levels. Inoculated and non-inoculated chickpea plants were grown at 23 °C in the glass house. Error bars indicate the standard error. Different letters and * represent statistically significant differences determined with ANOVA followed by Tukey’s test between transgenic and non-transgenic plants (n = 10, p ≤ 0.05).

Nitrogen fixation is one of the most energy expensive processes performed in plants^28,29^. Previously, we showed that *AtBAG4* expression supports higher photosynthetic levels in rice^20^. To investigate whether the increased nodulation in transgenic chickpea expressing *AtBAG4* was facilitated by access to increased energy resources, we measured and compared the photosynthetic capacity of the transgenic lines and non-transgenic controls. Like our rice data, the rhizobia inoculated chickpea lines expressing *AtBAG4* showed significantly higher net photosynthesis rates than the non-transgenic controls under irrigated conditions (Fig. 4). The non-inoculated transgenic chickpea plants are significantly stressed and therefore did not photosynthesise as much as their inoculated counterparts; we observed no significant differences between the non-inoculated transgenic lines and non-transgenic controls (Fig. 4).

### Nodule suppressive CLE peptides of chickpea are induced following *M*. *ciceri* infection

**Figure 5:**
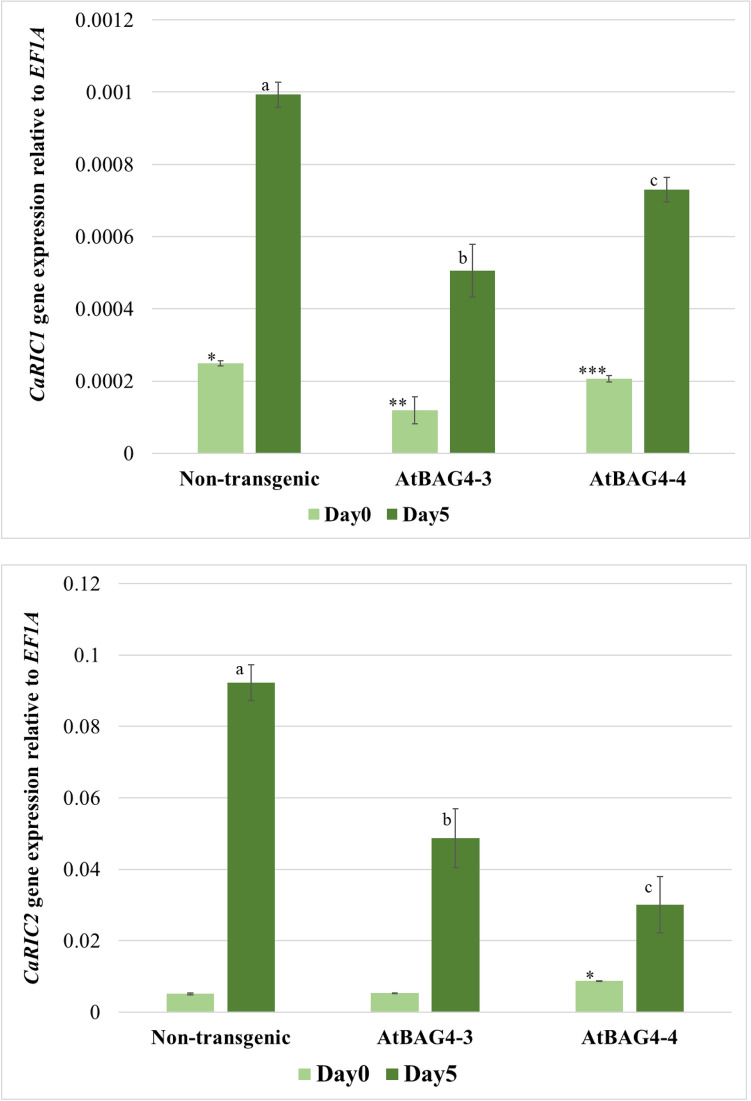
Expression of the nodulation CLE peptide genes, *CaRIC1* and *CaRIC2* in transgenic and non-transgenic chickpea. Values show the relative transcript abundance of *CaRIC1* and *CaRIC2* at 2 h and 5 days following the application of *M. ciceri*. Expression levels were determined using quantitative real-time reverse transcription PCR. Error bars indicate the standard error. Different letters and * represent statistically significant difference determined with ANOVA followed by Tukey’s test between transgenic and non-transgenic plants (n = 3, p ≤ 0.05).

To ensure a balance between nitrogen acquisition and energy expenditure, the AON pathway tightly regulates nodule formation and nitrogen fixation (Ferguson et al.^25^). In a negative feedback loop, nodule suppressing CLE peptides (*RIC1* and *RIC2*) are expressed in the root following rhizobial inoculation. CLE peptides have been identified in several model plants^5,25,30–33^, but there is little known about the nodulation-suppressing CLE peptides in chickpea. To evaluate whether *RIC* genes play a role in determining the nodulation of *AtBAG4* expressing chickpea, we analysed the expression levels of *CaRIC1* and *CaRIC2* after 2 h and 5 days of rhizobia inoculation. Expression levels of the housekeeping gene *EF1A*, were used for normalisation. Here, we observed that the *CaRIC1* and *CaRIC2* are induced by the compatible rhizobia strain *M. ciceri*, indicating that they are triggered during nodule organogenesis, with an expression pattern consistent with that reported for their respective orthologues in soybean and bean^32,34^. In both transgenic and non-transgenic plants, *CaRIC1* and *CaRIC2* gene expression levels were low 2 h post-inoculation and rose significantly by five days post-inoculation. *CaRIC2* expression was higher than *CaRIC1* after five days of rhizobia inoculation. Coinciding with high photosynthetic rates and increased nodule numbers, *CLE* gene expression in AtBAG4-3 and AtBAG4-4 plants was significantly lower than observed in non-transgenic control plants (Fig. 5).

## Discussion

Chickpea is one of the most widely grown legume crops worldwide, however, production is significantly hampered by drought and extreme temperatures (heat and frost at flowering)^35^. The suppression of plant PCD pathways can improve crop fitness in stressful environments, however, due to potentially deleterious effects on rhizobium symbiosis and nodulation, and plant development, doubts remain to whether PCD suppression is a suitable strategy to improve the agronomic performance of legumes. The Arabidopsis BAG family of co-chaperones is one of the few cell death regulators of plant cell death that are conserved in mammals and a potential target for biotechnological improvement of crops. Previous research has shown that transgenic expression of *AtBAG4* improves the agronomic performance of rice, tobacco and banana^18–20^. Although our knowledge of the molecular mechanisms driving AtBAG4-mediated tolerance remain incomplete, we recently revealed that AtBAG4 interacts with the selective autophagy substrate NBR1, and autophagy is required for AtBAG4-mediated stress tolerance (Under review). Here we used a combination of reverse genetics, expression analysis and stress assays to demonstrate that chickpeas expressing *AtBAG4* are drought tolerant, as evidenced by increased yields under stressed conditions. We also show that under well-watered conditions, transgenic *AtBAG4*-expressing chickpea formed nodules when inoculated with *M. ciceri* and fixed atmospheric nitrogen at similar or higher levels than the non-transgenic controls.

Contrary to expressing other cell death regulators, such as *CED-9* and *BI-1* in legumes, the expression of *AtBAG4* improved stress tolerance without affecting nodulation and nitrogen fixation detrimentally under well-watered conditions. The different outcome between AtBAG4 and other cell death suppressors may be due to the molecular roles that each of the proteins play and the fact that CED-9 and BI-1 increase autophagy. Although, autophagy is required for nodulation due to its role in host defence and cellular homeostasis^36,37^, modulating the pathways too much can have detrimental effects. For example, during nodule organogenesis, the host plant expresses nodulins to establish plant-rhizobia symbiosis^36^. Silencing of nodulins increases the expression of autophagy genes, including *Beclin-1*/*ATG6*, *ATG8*, *VPS15* and *PI3K,* leading to more defective nodules^36^. Conversely, autophagy is required for the symbiotic relationship between *P. vulgaris* and symbiotic microorganisms^37^. Downregulation of *Beclin1* in *P. vulgaris* results in less nodulation^37^.

In plants, the complete conservation of apoptosis pathways remains doubtful, however, plants expressing *CED-9* display reduced cell death and suppressed apoptotic-like hallmarks to suggest that mammalian apoptosis pathways are at least partially conserved in plants. Due to the lack of plant apoptotic-machinery, previous studies suggest that in plants, CED-9 suppresses cell death, by regulating autophagy pathways, via interaction with Beclin-1^38^. Similarly, Bax inhibitor 1 (BI-1) also interacts with ATG6/Beclin-1 in plants and positively regulates autophagy^39^. Expression of Bax-inhibitor I (*BI-I*) in common bean root increases the number of rhizobium infections and nodulation, however, the nodules die prematurely^40^. Given the vital role of autophagy within symbiotic relationships^36^, we hypothesise that the interaction of CED-9 and BI-1 with Beclin-1 and subsequent regulation of autophagy pathways may have a detrimental effect on nodule formation in legumes. In contrast, AtBAG4 is a co-chaperone potentially involved in chaperone-mediated selective autophagy. Although, autophagy pathways are involved in AtBAG4-mediated stress tolerance, AtBAG4 does not play a major regulatory role in plant autophagy pathways. This subtle difference between the roles of AtBAG4 and CED-9 and BI-1 may help explain why the expression of *AtBAG4* did not detrimentally affect on chickpea nodulation.

Potassium homeostasis is crucial to efficient regulation of stomatal movement. The proper regulation of potassium ion flux enables plants to respond rapidly to environmental conditions and prevent excessive water loss, specifically under drought and salinity conditions^41^. Previous research has shown a role for AtBAG4 in potassium homeostasis regulation via interaction with the potassium ion channel KAT1^21^. KAT1 help regulate potassium fluxes in the guard cell and to manipulate stomatal opening^42^. By directly interacting with KAT1, AtBAG4 positively regulates the KAT1 potassium channel to modulate stomatal movement^21^. The regulation of stomatal aperture has an essential role in drought stress tolerance and photosynthesis. Our results showed that *AtBAG4* expressing plants maintain higher photosynthetic levels under irrigated conditions, this is likely due to *AtBAG4* expression positively regulating stomatal aperture, thus facilitating efficient gas exchange to optimise the compromise between photosynthesis and water management. Although not tested in this study, the better photosynthetic control may also allow the *AtBAG4* expressing plants to better manage stress tolerance and nodulation. Biological nitrogen fixation and nodule formation are resource-intensive processes^28,43^. Irrigated *AtBAG4* expressing transgenic chickpea plants produce more energy via higher net photosynthesis to support nodulation possibly via regulation of AON pathway. During subsequent drought stress, these resources may better support drought stress tolerance. The increased energy capacity in transgenic plants helps to improve the water use efficiency under drought stress therefore, it prevents excessive water loss and provides drought stress tolerance.

Legume nodulation is regulated by systemic mechanisms and local host defence pathways in response to rhizobia interaction. In contrast, the premature cell death observed following *AtBI-I* expression, is thought to occur due to activation of plant defence systems that induce plant cell death^40,44^. The cell death response in *BI-1* expressing plants may also be attributed to BI-1’s interaction with Beclin-1 which is an established player in the hypersensitive response of tobacco to infection with Tobacco mosaic virus (TMV)^45^.

**Figure 6:**
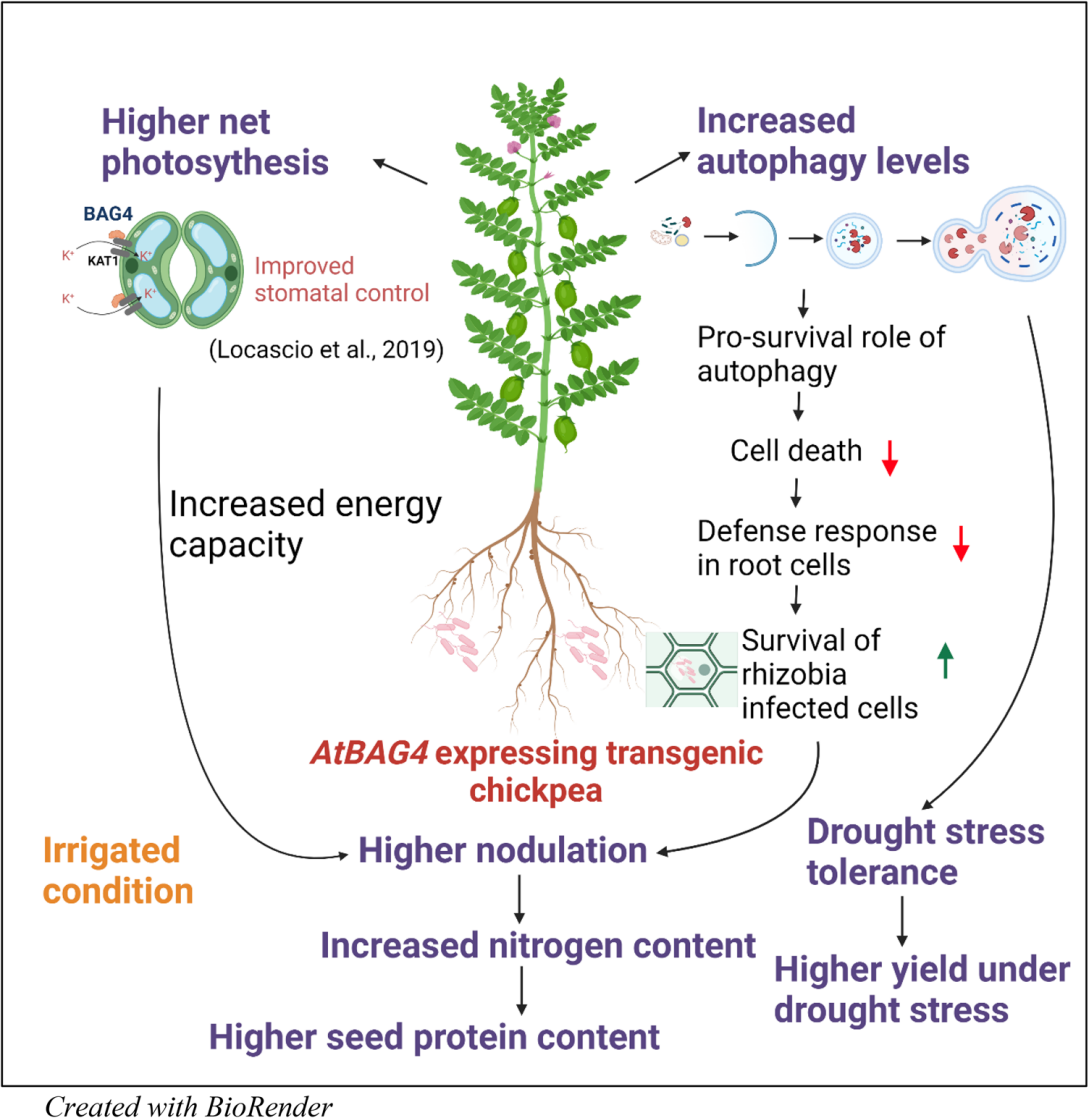
Model of how AtBAG4 confers drought stress tolerance in chickpea without affecting yield and nodulation. Transgenic chickpea expressing *AtBAG4* showed higher photosynthesis under well-watered conditions. Locascio et al. showed that BAG4 directly binds with KAT1 and aids in K^+^ fluxes in the guard cells and results in regulating the stomatal opening^21^. Higher nodulation leads to higher nitrogen content in transgenic shoots and accumulated in seeds as indicated by higher seed protein content in transgenic lines. Increased nodulation under full irrigated conditions was supported by high energy capacity in transgenic chickpea plants. Another possible reason for increased nodulation in transgenic plants is the modulation of defence response upon rhizobia infection. Higher autophagy levels in *AtBAG4* overexpressing plants suppress cell death and defence response in roots. Therefore, more rhizobia infected cells will survive to form more nodules. Increased nodulation leads to increased nitrogen content in the shoot under nitrogen depleted conditions. Therefore, At*BAG4* expression potentially increases the nitrogen use efficiency in transgenic chickpea plants. Furthermore, our results showed that there is no yield penalty in rhizobia inoculated *AtBAG4* expressing transgenic chickpea lines under well-watered conditions. Overall, this study suggests that transgenic expression of *AtBAG4* in chickpea a good strategy to improve chickpea cultivation in future.

The elicitation of the plant defence limits the number of infection events in the roots and therefore contributes to determining the number of nodules formed in the legume root. During the rhizobia infection, roots cells may identify some rhizobia as pathogens. A universal response of plants to pathogen attack is an oxidative burst and cell death at the site of pathogen penetration^46^. By improving stress tolerance and ROS detoxification, *AtBAG4* expression may help attenuate cell death associated with plant defence to allow more rhizobia to survive and subsequently increase nodulation. Specifically, in combination with autophagy pathways, AtBAG4 may help suppress the accumulation of unfolded proteins, which can result in cell death if left unchecked. By reducing the levels of cell death associated with rhizobia infection, AtBAG4 subsequently supports increased nodulation (Fig. 6). Increased nodule numbers lead to higher shoot nitrogen content in rhizobia inoculated transgenic chickpea (Fig. 6).

## Conclusion and future perspectives

We showed that expression of *AtBAG4*, a cytoprotective co-chaperone, is a promising strategy to improve legume stress tolerance while not affecting nodulation, nitrogen fixation or yield under well-watered conditions. Further studies are however needed to understand the effect of *AtBAG4* expression on chickpea nodulation, net photosynthesis, and nitrogen fixation under water-deficit stress. Future studies should also investigate the relationship between, nodule formation, nodule senescence and CLE peptide signalling in chickpea, potentially leading to advances in improving chickpea nodulation in agriculture.

## Materials and methods

### Plant growth conditions

Independent transgenic chickpea lines of the commercially available HatTrick cultivar constitutively expressing a single copy of the *AtBAG4* transgene were generated previously by Tropical Pulses for Queensland (TPFQ) project at the Queensland University of Technology and the transgenic seeds for this study were kindly provided by TPFQ team ^47^. Transgenic chickpea seeds were surface sterilised with 1% sodium hypochlorite for three minutes with vigorous shaking. Following sterilisation, the seeds were thoroughly rinsed four to five times with sterile milliQ water and placed on plates containing sterile wet filter paper for germination at 23 °C in the dark. Three days post-germination, the seedlings were placed in 4L pots containing grade 3 sterile vermiculite (for nodulation assay) or soil (for yield assay). Plants were watered (200 mL) every second day and supplemented with nitrogen-free B & D solution once a week^48^. Unless otherwise stated, all plants were grown at 23 °C for 16 h light and 8 h dark cycle with 40–60% relative humidity. Non-transgenic chickpea was used as controls.

### Bacterial growth conditions

For nodulation, *Mesorhizobium ciceri* strain CC1192 was grown for 48 h at 28 °C in Yeast Mannitol Broth (YMB), harvested and diluted with nitrogen-free B&D solution to a final concentration of OD_600_ ~ 0.1^49^. Each pot was treated with 150 mL of diluted *M.ciceri*. As controls, a complete set of non-inoculated plants from each genotype, including non-transgenic controls, was supplied with nitrogen-free B&D solution.

### Drought stress assay

All drought experiments were conducted in the PC2 (Physical containment level 2) glasshouse at QCDF Redland, Queensland, Australia. Non-transgenic and three transgenic chickpea lines were planted in pots (165 mm diameter × 240 mm height) containing 4 kg of soil (1:1 potting mix and sand) with a randomised statistical block design. A total of three replicates with three seedlings per replicate (total nine plants) of each line were planted in pots for the drought treatments and irrigated controls. All plants were irrigated with 200 mL water three times per a week to maintain the soil relative water content above 50% of field capacity until early flowering stage (47 days post-sowing). For drought treatment, the plants were not watered after the 47th day until senescence. As irrigated controls, plants were supplied 200 ml of water three times per week until pod maturity before withholding irrigation completely for pod drying. The number of seeds per plant and 100 seed weight of each line were counted under drought stress and irrigated conditions.

### Nodulation assay

Three-day old seedlings were transferred to pots containing sterile vermiculite and treated with 150 mL of *M. ciceri* inoculum. Five plants were grown in each pot and five biological replicates were assessed for each genotype. Triplicate experiments were performed. Plants were grown for 21 days before nodule harvesting and recording of the number of nodules per plant. Plants were watered every second day with equal amounts of water added to each pot. The plants were dried separately at 60 °C for two days before measuring the dry weights.

### Light microscopy

Nodules were harvested after 21 days, fixed in 0.05% paraformaldehyde, and stored at 4 °C in 70% ethanol until sectioning. The nodules were processed for five hours and twenty minutes using a Leica ASP300S tissue processor before embedding into paraffin. Samples were bisected longitudinally through the nodules to obtain cross sections of both the nodule and the root. These were placed face down in the cassette (Thermofisher Shandon Histocentre 3 Embedding Station). Thin nodule sections were made using Leica RM2245 microtome followed by heat fixing for 40 min at 60 °C. Following fixation, the slides were stained with 0.1% Toluidine Blue O and scanned using a 3D Histech Panoramic Scan II on the 40X objective. For the detailed procedure see Supplementary Table 1.

### Isotope analysis

*M. ciceri*-inoculated and non-inoculated chickpea shoots (2 months old) were harvested, dried for two days at 60 °C and ground with a planetary cylinder mill. The samples were analysed for N content and delta ^15^N signatures using an Isotope ratio mass spectrometer IRMS (20–22 Sercon Limited, UK). Five biological replicates were analysed from each genotype.

### Net photosynthesis measurements

Inoculated and non-inoculated chickpea plants grown in the glasshouse were used for the photosynthesis measurements. Net photosynthesis was measured using a LiCOR Infra-Red Gas Analyser LI-6400 XT (John Morris Scientific, Chatwood, NSW, Australia) according to the manufacturer’s instructions. The third newest fully grown compound leaf of each plant was used for the measurement and all the measurements were taken before flowering. Ten biological replicates were used for each genotype.

### RNA extraction and cDNA synthesis

For CLAVATA/ESR-related (CLE) peptide analysis, three-day old seedlings were planted in pots containing sterile vermiculite and grown for five days. Plants were watered every second day with sterile distilled water. *M. ciceri* inoculum was added to each pot eight days post-germination^34^. The entire root system was harvested 0 and 5 days post-inoculation, snap frozen in liquid nitrogen and stored at − 80 °C until RNA extraction using a plant RNeasy kit (Qiagen) according to the manufacturer’s instructions. To remove contaminating genomic DNA from the sample, approximately 1 μg of total RNA was treated with 1U of RQ1 DNase (Promega) at 37 °C for 30 min. The DNase reaction was terminated by the addition of 1μL of RQ1 DNase stop solution and incubation at 65 °C for 10 min. cDNA was synthesized using a GoScript Reverse transcription system (Promega) according to the manufacturer’s instructions.

For *AtBAG4* gene expression analysis, leaf samples were collected from two month old glasshouse grown plants and snap frozen. RNA was isolated using plant RNeasy kit according to the manufacturer’s instructions similar procedure was followed as above.

### Quantitative real time PCR

Quantitative real time PCR was performed according to the methods described in Hayashi et al.^50^. Primers for *AtBAG4*, *CaRIC1* and *CaRIC2* genes were designed using Primer3 bioinformatic software (MIT) software^51^. A list of primers used in this study is provided in Table S2. *CaEF1A* was used as a housekeeping gene to normalise the *AtBAG4*, *CaRIC1* and *CaRIC2* transcript abundance.

### Statistical analysis

All data were subjected to one-way ANOVA (MINITAB 18 Statistical Software). Differences among means for treatments were evaluated by Tukey pairwise comparison test at 0.05 probability levels.

All the methods were carried out in accordance with Queensland University of Technology guidelines and regulations.

### Supplementary Information

Supplementary Information.

## Data Availability

All data generated or analysed during this study are included in this published article (and its supplementary information files).

## References

[1] Summo C (2019). Nutritional, physico-chemical and functional characterization of a global chickpea collection. J. Food Compos. Anal..

[2] Gaur PM, Tuteja N, Gill SS (2013). Climate change and heat stress tolerance in chickpea. Climate Change and Plant Abiotic Stress Tolerance.

[3] Devasirvatham V, Tan DK (2018). Impact of high temperature and drought stresses on chickpea production. Agronomy.

[4] Stagnari F, Maggio A, Galieni A, Pisante M (2017). Multiple benefits of legumes for agriculture sustainability: An overview. Chem. Biol. Technol. Agric..

[5] Mens C (2021). Characterisation of *Medicago*
*truncatula* CLE34 and CLE35 in nitrate and rhizobia regulation of nodulation. New Phytol..

[6] Roy SJ, Tucker EJ, Tester M (2011). Genetic analysis of abiotic stress tolerance in crops. Curr. Opin. Plant Biol..

[7] Mittler R (2006). Abiotic stress, the field environment and stress combination. Trends Plant Sci..

[8] Kabbage M, Kessens R, Bartholomay LC, Williams B (2017). The life and death of a plant cell. Annu. Rev. Plant Biol..

[9] Chen S, Dickman MB (2004). Bcl-2 family members localize to tobacco chloroplasts and inhibit programmed cell death induced by chloroplast-targeted herbicides. J. Exp. Bot..

[10] Mitsuhara I, Malik KA, Miura M, Ohashi Y (1999). Animal cell-death suppressors Bcl-xL and Ced-9 inhibit cell death in tobacco plants. Curr. Biol..

[11] Qiao J (2002). Enhanced resistance to salt, cold and wound stresses by overproduction of animal cell death suppressors Bcl-xL and Ced-9 in tobacco cells—Their possible contribution through improved function of organella. Plant Cell Physiol..

[12] Xu P, Rogers SJ, Roossinck MJ (2004). Expression of antiapoptotic genes bcl-xL and ced-9 in tomato enhances tolerance to viral-induced necrosis and abiotic stress. Proc. Natl. Acad. Sci..

[13] Shabala S, Cuin TA, Prismall L, Nemchinov LG (2007). Expression of animal CED-9 anti-apoptotic gene in tobacco modifies plasma membrane ion fluxes in response to salinity and oxidative stress. Planta.

[14] Thanthrige N (2020). Centrality of BAGs in plant PCD, stress responses, and host defense. Trends Plant Sci..

[15] Dickman M (2001). Abrogation of disease development in plants expressing animal antiapoptotic genes. Proc. Natl. Acad. Sci..

[16] Robert G, Muñoz N, Melchiorre M, Sanchez F, Lascano R (2014). Expression of animal anti-apoptotic gene Ced-9 enhances tolerance during Glycine max L.-*Bradyrhizobium*
*japonicum* interaction under saline stress but reduces nodule formation. PloS One.

[17] Kabbage M, Dickman MB (2008). The BAG proteins: A ubiquitous family of chaperone regulators. Cell Mol. Life Sci..

[18] Doukhanina EV (2006). Identification and functional characterization of the BAG protein family in *Arabidopsis*
*thaliana*. J. Biol. Chem..

[19] Namukwaya B (2015). Evaluation of Transgenic Bananas Expressing Anti-Apoptotic Genes for Resistance Against Fusarium wilt.

[20] Hoang TM (2015). Development of salinity tolerance in rice by constitutive-overexpression of genes involved in the regulation of programmed cell death. Front. Plant Sci..

[21] Locascio A (2019). BCL2-associated athanogene4 regulates the KAT1 potassium channel and controls stomatal movement. Plant Physiol..

[22] Ulbricht A (2015). Induction and adaptation of chaperone-assisted selective autophagy CASA in response to resistance exercise in human skeletal muscle. Autophagy.

[23] Rosati A, Graziano V, De Laurenzi V, Pascale M, Turco M (2011). BAG3: A multifaceted protein that regulates major cell pathways. Cell Death Dis..

[24] Behl C (2016). Breaking BAG: The co-chaperone BAG3 in health and disease. Trends Pharmacol. Sci..

[25] Ferguson BJ (2019). Legume nodulation: The host controls the party. Plant Cell Environ..

[26] Chalk PM (1985). Estimation of N2 fixation by isotope dilution: An appraisal of techniques involving 15N enrichment and their application. Soil Biol. Biochem..

[27] Unkovich M (2008). Measuring Plant-Associated Nitrogen Fixation in Agricultural Systems.

[28] Oldroyd GE (2013). Speak, friend, and enter: Signalling systems that promote beneficial symbiotic associations in plants. Nat. Rev. Microbiol..

[29] Ferguson BJ (2010). Molecular analysis of legume nodule development and autoregulation. J. Integr. Plant Biol..

[30] Okamoto S (2009). Nod factor/nitrate-induced CLE genes that drive HAR1-mediated systemic regulation of nodulation. Plant Cell Physiol..

[31] Mortier V (2010). CLE peptides control *Medicago*
*truncatula* nodulation locally and systemically. Plant Physiol..

[32] Reid DE, Ferguson BJ, Hayashi S, Lin Y-H, Gresshoff PM (2011). Molecular mechanisms controlling legume autoregulation of nodulation. Ann. Bot..

[33] Ferguson BJ, Mathesius U (2014). Phytohormone regulation of legume-rhizobia interactions. J. Chem. Ecol..

[34] Ferguson BJ (2014). The soybean (Glycine max) nodulation-suppressive CLE peptide, Gm RIC 1, functions interspecifically in common white bean (Phaseolus vulgaris), but not in a supernodulating line mutated in the receptor Pv NARK. Plant Biotechnol. J..

[35] Jha UC (2014). Abiotic stresses, constraints and improvement strategies in chickpea. Plant Breed..

[36] Rodríguez-López J, López AH, Estrada-Navarrete G, Sánchez F, Díaz-Camino C (2019). The noncanonical heat shock protein Pv Nod22 is essential for infection thread progression during rhizobial endosymbiosis in common bean. Mol. Plant-Microbe Interact..

[37] Estrada-Navarrete G (2016). An autophagy-related kinase is essential for the symbiotic relationship between *Phaseolus*
*vulgaris* and both rhizobia and arbuscular mycorrhizal fungi. Plant Cell.

[38] Takacs-Vellai K (2005). Inactivation of the autophagy gene bec-1 triggers apoptotic cell death in *C*. *elegans*. Curr. Biol..

[39] Xu G (2017). Plant bax inhibitor-1 interacts with ATG6 to regulate autophagy and programmed cell death. Autophagy.

[40] Hernández-López A (2019). Uncovering bax inhibitor-1 dual role in the legume–rhizobia symbiosis in common bean roots. J. Exp. Bot..

[41] Lebaudy A (2008). Plant adaptation to fluctuating environment and biomass production are strongly dependent on guard cell potassium channels. Proc. Natl. Acad. Sci..

[42] Nakamura RL (1995). Expression of an *Arabidopsis* potassium channel gene in guard cells. Plant Physiol..

[43] Ferguson B, Lin M-H, Gresshoff PM (2013). Regulation of legume nodulation by acidic growth conditions. Plant Signal. Behav..

[44] Wang C (2016). Nodules with activated defense 1 is required for maintenance of rhizobial endosymbiosis in *Medicago*
*truncatula*. New Phytol..

[45] Liu Y (2005). Autophagy regulates programmed cell death during the plant innate immune response. Cell.

[46] Balint-Kurti P (2019). The plant hypersensitive response: Concepts, control and consequences. Mol. Plant Pathol..

[47] Das Bhowmik SS (2019). Robust genetic transformation system to obtain non-chimeric transgenic chickpea. Front. Plant Sci..

[48] Broughton W, Dilworth M (1971). Control of leghaemoglobin synthesis in snake beans. Biochem. J..

[49] Mandal D, Sinharoy S (2019). A toolbox for nodule development studies in chickpea: A hairy-root transformation protocol and an efficient laboratory strain of *Mesorhizobium* sp. Mol. Plant-Microbe Interact..

[50] Hayashi S (2012). Transient nod factor-dependent gene expression in the nodulation-competent zone of soybean (Glycine max [L.] Merr.) roots. Plant Biotechnol. J..

[51] Untergasser A (2012). Primer3—New capabilities and interfaces. Nucleic Acids Res..

