# Supplementary material for: The cytoprotective co-chaperone, AtBAG4, supports increased nodulation and seed protein content in chickpea without yield penalty
Source: Sci Rep. 2023 Oct 29;13:18553. doi: 10.1038/s41598-023-45771-3 (PMC10613627; doi:10.1038/s41598-023-45771-3)
Supplement: Supplementary file 1 — Supplementary Information. [file 41598_2023_45771_MOESM1_ESM.docx]

**Supplementary Information**

**
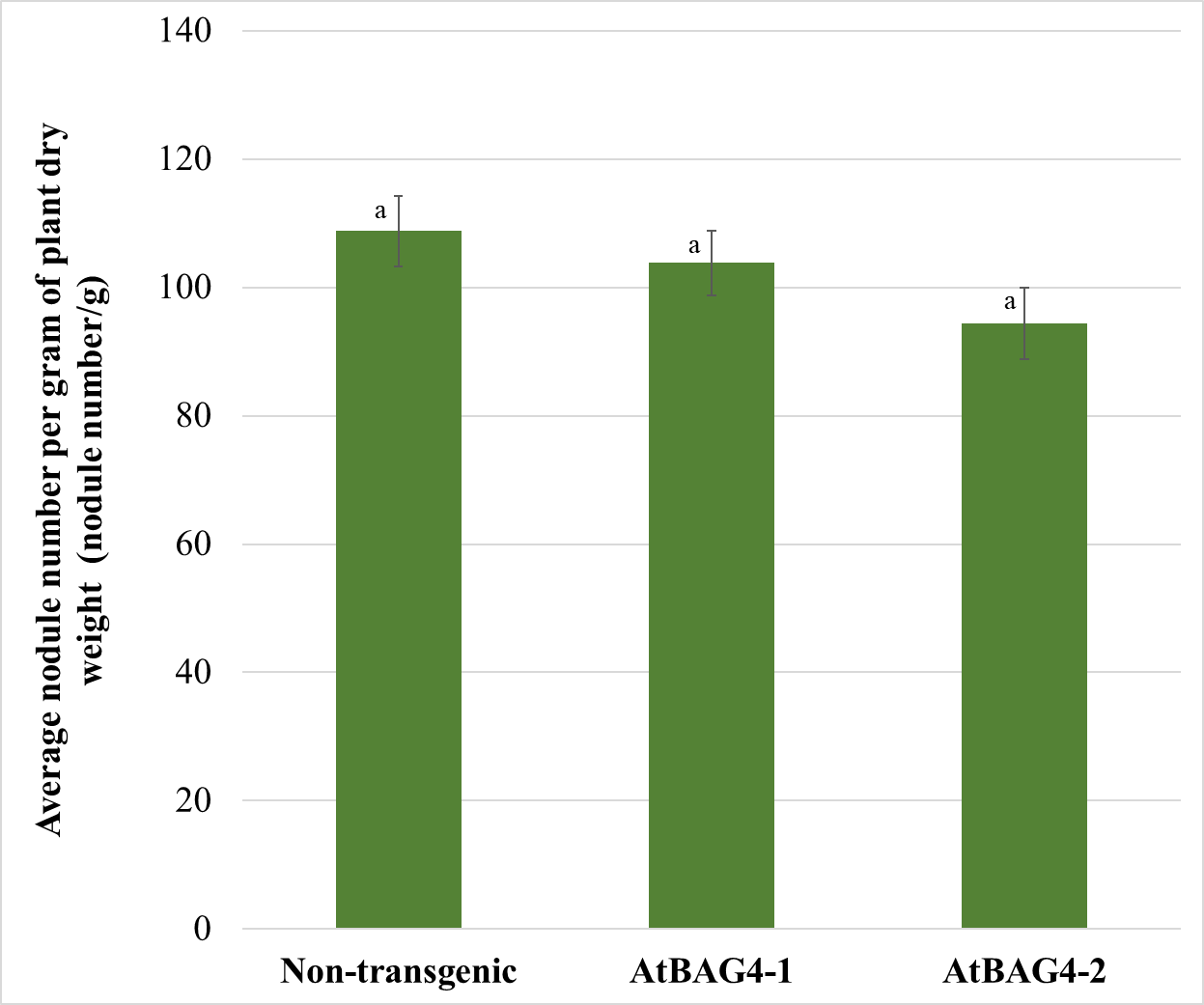
**

**Supplementary Fig. 1** Transgenic lines AtBAG4-1 and AtBAG4-2 showed similar nodulation as the non-transgenic control under well watered conditions. Error bars indicate the standard error. Different letters represent statistically significant differences determined with ANOVA followed by Tukey’s test between transgenic and non-transgenic plants (n=5, p ≤ 0.05).


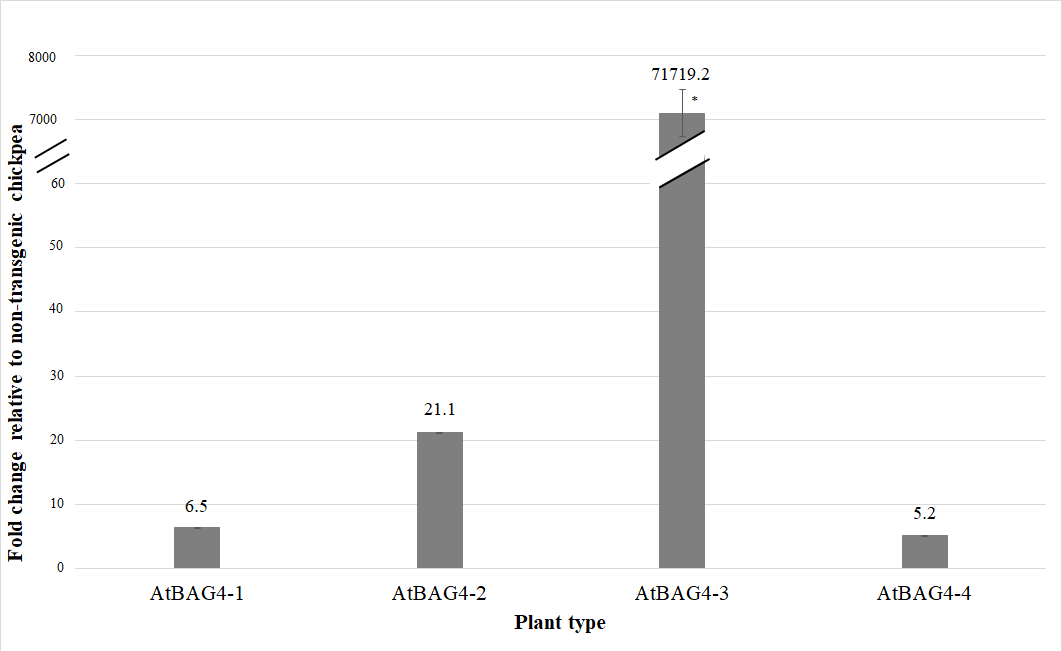


**Supplementary Fig. 2** *AtBAG4* gene expression levels relative to non-transgenic chickpea. *CaEF1A* was used as a housekeeping gene. Error bars indicate the standard error. Asterisk symbol (*) represents statistically significant difference was determined with ANOVA followed by Tukey’s test between transgenic and non-transgenic plant (n=3, p ≤ 0.05).


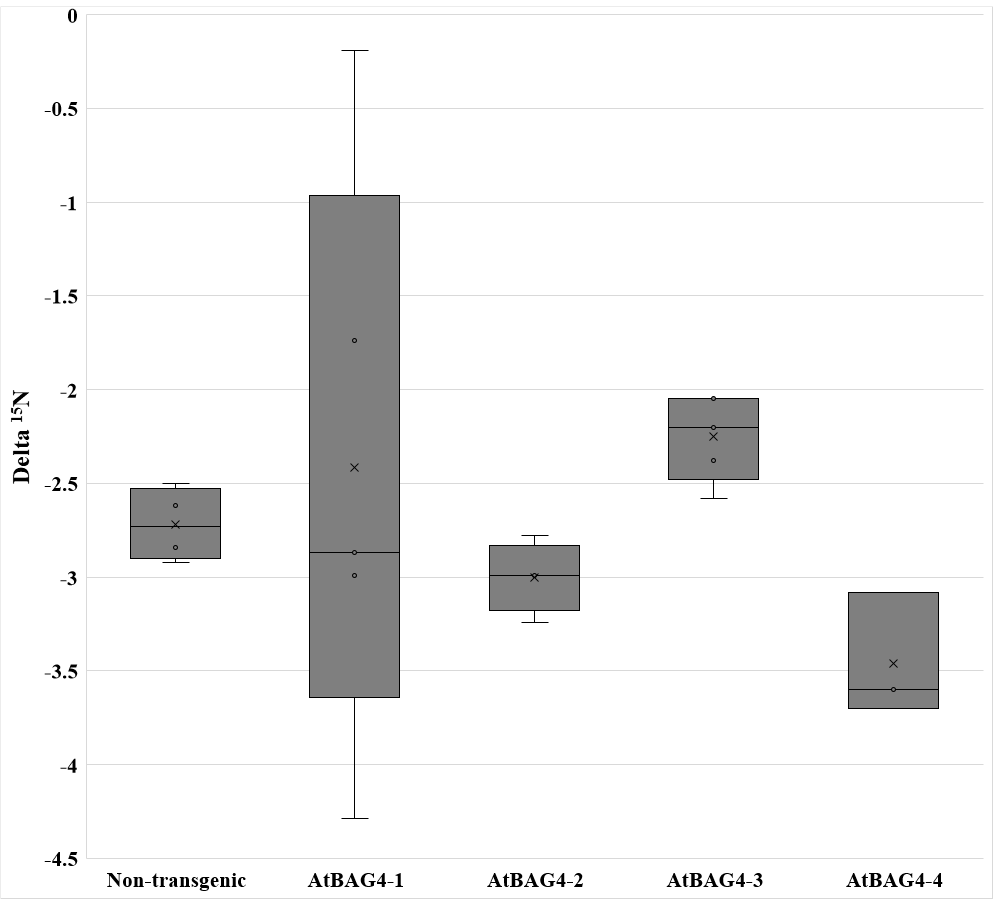


**Supplementary Fig. 3** Transgenic chickpea fixes atmospheric nitrogen. Delta ^15^N values were recorded. The data were statistically analysed with ANOVA followed by Tukey’s test. (n=5, p ≤ 0.05). Error bars indicate the standard error.


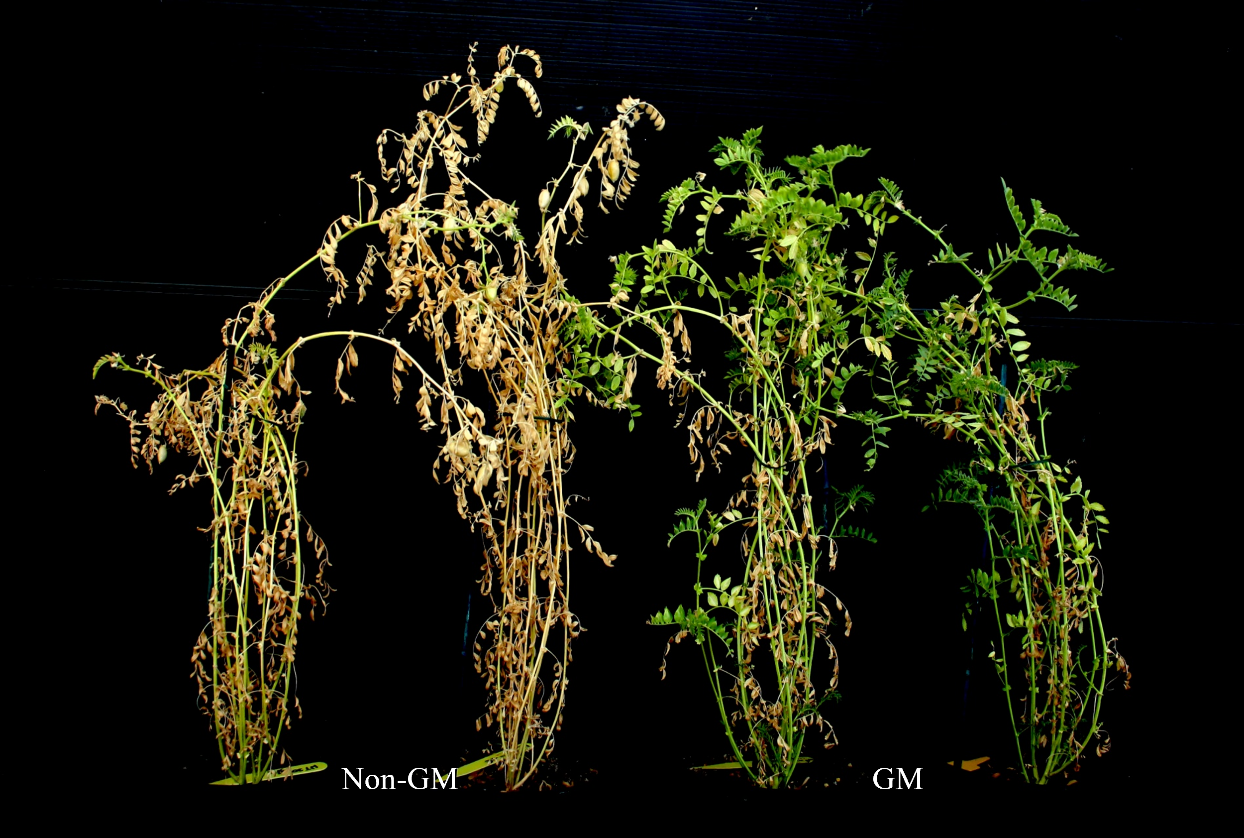
**Supplementary Fig.4** Plant morphology of non-transgenic (left) and transgenic (right) chickpea under water-deficit conditions.

**Supplementary Table 1** Procedure for nodule sectioning, staining and microscopic observation

| **Processing** | **Instrument** | Leica ASP300S Tissue Processor |
| --- | --- | --- |
|  | **Protocol** | ‘Short Run’  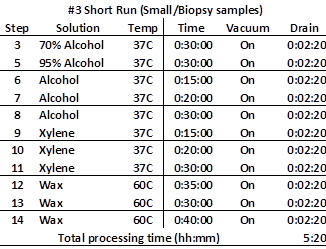 |
| **Embedding** | **Instrument** | Thermofisher Shandon Histocentre 3 Embedding Station |
|  | **Protocol** | Standard paraffin embedding procedure. Samples were bisected longitudinally through the nodules to obtain cross sections of both the nodule and root. These were placed faced down in the cassette. |
| **Microtomy** | **Instrument** | Leica RM2245 |
|  | **Protocol** | Five slides were collected per paraffin block.  Where possible (dependent upon block size/ amount of nodules present), two sections of tissue were collected onto each slide.  ICON+ slides were used.  Slides were heat fixed after sectioning, 40 min, 60 ^0^C. |
| **Staining**  (Two slides were stained for each paraffin block – one for H&E, one for Tol. blue) | **H&E (Automated)** | **Instrument**  Leica Autostainer XL with attached Leica Robotic Coverslipper CV5030 and transfer arm.  **Reagents/Solutions**  Harris Haematoxylin (POCD, HHXIMP)  Aqueous Eosin (Amber Scientific, EOA1)  **Protocol**  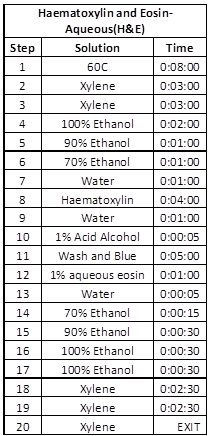 |
|  | **Toluidine Blue**  **(done by hand)** | **Reagents**  Toluidine blue O (Sigma Aldrich, 198161)  **Solutions**  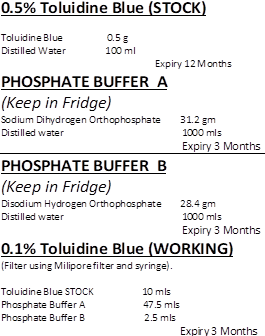  **Protocol**  1. Deparaffinise section to water using Leica Autostainer.  2. Flood slides with 0.1% Tol blue working solution for 1 minute.  3. Rinse slide in tap water for 10 seconds.  4. Dehydrate through alcohols and xylene, coverslip. |
| **Microscopy** | **Instrument** | 3D Histech Panoramic SCAN II  Scanned on the 40x objective. |

**Supplementary Table 2** Primers used in this study

| Primer number | Oligonucleotides | Purpose |
| --- | --- | --- |
| P1 | TGACGGTCGAAGACTGAACA | *CaRIC1* Forward |
| P2 | CCAGGTGAAAGACGATCTCC | *CaRIC1* Reverse |
| P3 | TCTTGACTTTGCAAGCTCGT | *CaRIC2* Forward |
| P4 | GGCCTCCTGGTGAGAGTCTA | *CaRIC2* Reverse |
| P5 | GTGGTTTTGAGGCTGGTATCTC | *EF1A* Forward |
| P6 | GGCCTTTGAGTACTTGGGTGTA | *EF1A* Reverse |
| P7 | GCTGAAGGGGACGCTAAAG | *AtBAG4* Forward |
| P8 | TTTGCTCTGATCCACAAACG | *AtBAG4* Reverse |
| P9 | TGGTGGTATGCTCGTCCAAC | *AtBAG4* Forward |
| P10 | GAGGGTTCAAGCTTCCGACA | *AtBAG4* Reverse |

P1- P8 - Expression analysis

P9 and P10 - PCR screening
